# Improved Adaptive Successive Cancellation List Decoding of Polar Codes

**DOI:** 10.3390/e21090899

**Published:** 2019-09-17

**Authors:** Xiumin Wang, Jinlong He, Jun Li, Zhuoting Wu, Liang Shan, Bo Hong

**Affiliations:** 1 College of Information Engineering, China Jiliang University, Hangzhou 310018, China; 05a0303091@cjlu.edu.cn (X.W.); S1803081215@cjlu.edu.cn (J.H.); P1603085219@cjlu.edu.cn (Z.W.); hongbo@cjlu.edu.cn (B.H.); 2 Binjiang College, Nanjing University of Information Science & Technology, Wuxi 214105, China

**Keywords:** polar codes, SC decoding, SCL decoding algorithm, AD-SCL algorithm, segmentation decoding algorithm

## Abstract

Although the adaptive successive cancellation list (AD-SCL) algorithm and the segmented-CRC adaptive successive cancellation list (SCAD-SCL) algorithm based on the cyclic redundancy check (CRC) can greatly reduce the computational complexity of the successive cancellation list (SCL) algorithm, these two algorithms discard the previous decoding result and re-decode by increasing *L*, where *L* is the size of list. When CRC fails, these two algorithms waste useful information from the previous decoding. In this paper, a simplified adaptive successive cancellation list (SAD-SCL) is proposed. Before the re-decoding of updating value *L* each time, SAD-SCL uses the existing log likelihood ratio (LLR) information to locate the range of burst error bits, and then re-decoding starts at the incorrect bit with the smallest index in this range. Moreover, when the segmented information sequence cannot get the correct result of decoding, the SAD-SCL algorithm uses SC decoding to complete the decoding of the subsequent segmentation information sequence. Furthermore, its decoding performance is almost the same as that of the subsequent segmentation information sequence using the AD-SCL algorithm. The simulation results show that the SAD-SCL algorithm has lower computational complexity than AD-SCL and SCAD-SCL with negligible loss of performance.

## 1. Introduction

The polar codes [1], as a channel coding technique for 5G communication, are used as the coding of the control channel for transmitting signaling or synchronizing data. Polar codes become the research hotspot. In decoding, the SC decoding algorithm based on polarization characteristics of the channel proposed by Professor Arikan is a suitable decoding algorithm for polar codes. Based on this decoding algorithm, polar codes have been proved capable of reaching the Shannon limit when the code length approaches infinity. Later, Tian et al. proposed a scenario-simplified successive cancellation decoding algorithm based on the erasure channel, which can simplify the calculation operations in the decoding process [2].

However, SC decoding has some disadvantages, such as large decoding delay and strong inter-symbol interference, which make SC decoding performance worse in the case of limited code length. In order to improve the decoding performance in the SC algorithm, the SCL decoding algorithm proposed in [3] uses a binary tree to represent the multiple possibilities of the SC algorithm in bitwise decoding and retains L candidate decoding paths, where *L* is the size of list. This method can effectively reduce the inter-symbol interference and improve the decoding performance of polar codes. Subsequently, Niu et al. proposed a CA-SCL algorithm, which selects reliable paths of SCL in [4] through the cyclic redundancy check aided (CRC-aided) decoding of polar codes. The algorithm further improves the performance of SCL, and when *L* is big enough, the performance of the algorithm can approach the maximum likelihood probability. In [5], a successive cancellation priority (SCP) decoding algorithm was proposed to reduce the decoding delay by avoiding the expansion of some unnecessary paths.

In [6], a new SCL decoding was proposed by using multiple CRC codes, which can improve the performance of decoding. However, using multiple CRC codes increases memory and time complexity, which can be reduced by optimizing CRC positions in combination with a modified decoding operation. Cheng et al. proposed the bit-flip algorithm for the CA-SCL algorithm, which can improve the decoding performance of polar codes compared to CRC-aided decoding of polar codes [7]. Although the SCL decoding algorithm has excellent decoding performance by adding CRC, its memory complexity and time complexity increase with the increase of L.

The method of reducing the complexity in SCL decoding is mainly divided into two types. On the one hand, the computational parallelism of SCL is improved and the computational process is simplified to reduce the time complexity of SCL decoding. In [8], a method for estimating path metrics (PM) by using Rate-1 and SPC nodes for the SSC and SCL algorithm in parallel was proposed. This method greatly reduces the time complexity of the SCL algorithm, but it brings about the uncertainty of the estimation results and leads to performance loss. Based on this, the simplified successive cancellation list (SSCL) algorithm proposed in [9] can identify and simplify the redundancy calculation in SCL. This method greatly reduces the time complexity without loss of performance.

On the other hand, it is the optimization of path management in SCL decoding. Zhou et al. proposed the segmented CRC-aided successive cancellation list (SCA-SCL) decoding technique which can reduce the complexity of decoding [10]. Path selection can also be optimized. In [11,12,13,14,15], different schemes for optimizing L paths were proposed. In order to reduce the complexity of SCL, Yang et al. proposed a more convenient method to select L paths from 2L paths based on RS sorting [16], which reduces the complexity of selecting paths in SCL from $O\left(NL\log_{2}L\right)$ to O(NL).

In [17], they proposed a method to simplify the number of paths retained in the SCL decoding process according to the transition probability, which realizes SCL decoding with variable L and also reduces the complexity.

Hashemi et al. proposed partitioned SCL (PSCL) decoding technique which can reduce the required memory [18]. Although the method above reduces the complexity of SCL to a certain extent, they all have a certain impact on the performance of SCL. Based on the PSCL algorithm, the generalized PSCL (GPSCL) algorithm proposed by Hashemi et al. can improve performance under the same complexity [19].

Li et al. put forward the adaptive SCL (AD-SCL) decoding algorithm [20], which reduces the complexity of SCL in the case of specified performance. The difference between AD-SCL and CA-SCL lies in that AD-SCL sets the minimum path value and the maximum path value, while SCL starts to decode from $L_{\min}$. If there is an error decoding result after CRC, re-decoding starts by increasing L until the decoding passes the CRC or L equals to $L_{\max}$.

Although the algorithm reduces the computation complexity without loss of performance, when a CRC fails, it has to re-decode from the first bit, which greatly increases the decoding delay caused by error bits.

Recently, Wang et al. proposed a segmented adaptive SCL decoding algorithm (SCAD-SCL) based on AD-SCL in [21]. This algorithm divides codewords into two segments by adding CRC in the middle of the codewords and uses the AD-SCL method in decoding. Hence, when decoding errors from the second part occur, there is no need to re-decode from the first bit.

Compared with AD-SCL, SCAD-SCL reduces computation complexity. However, both AD-SCL and SCAD-SCL discard the previous decoding results and directly re-decode by increasing L when CRC fails, which wastes the useful information from the previous decoding. In addition, the experiment analysis shows that the performance of the second part is almost independent of size L, when there is an error in the first part of the decoding. Based on the above analysis, SCAD-SCL proposed in [21] still needs to be improved further. In this paper, a simplified adaptive SCL segmentation algorithm (SAD-SCL) based on CRC-aided is proposed. Before the re-decoding of updating value *L* each time, SAD-SCL uses the existing log likelihood ratio (LLR) information to locate the range of burst error bits. This proposed method is to locate a starting bit in the re-decoding. Then, the re-decoding starts at the locating bit with the smallest index in this range. The SAD-SCL algorithm further avoids redundant computation in the decoding process with negligible loss of performance and has a lower computational complexity than SCAD-SCL.

## 2. Theory of Polar Codes

A method for constructing polar codes based on channel polarization is given in [1]. The construction of the polar codes can be defined by $\left(N,K,\Lambda ,u_{\Lambda^{C}}\right)$, where N denotes the code length, K denotes the number of information bits, the code rate is K/N, Λ denotes the set of the index of the information bits, $u_{\Lambda }$ denotes the set of the information bits, and $u_{\Lambda^{C}}$ denotes the frozen bits. the frozen bits are usually set to zero. $u_{1}^{N}$ denotes the information sequence before encoding, and then the encoding process can be expressed as

(1) $$x_{1}^{N}=u_{1}^{N}G_{N}$$

where $x_{1}^{N}$ is the encoding sequence, $G_{N}$ is the generator matrix of polar codes. $G_{N}$ is represented by

(2) $$G_{N}=B_{N}F^{⊗n}$$

where $n=log_{2}N$, $F^{⊗n}$ denotes n-order Kronecker power, and $F=\left[\begin{array}{cc} 1 & 0\\ 1 & 1 \end{array}\right]$.

The decoding of polar codes can be regarded as the process of channel splitting, and the transition probability of each sub-channel is related to each other. For the information bit i, the hard decision value ${\hat{u}}_{i}$ is calculated by the received sequence $y_{1}^{N}$ and the sequence ${\hat{u}}_{1}^{i-1}$ that has been decoded previously. The specific calculation formula is [1]

(3) $$\delta \left(y_{1}^{N},{\hat{u}}_{1}^{i-1}\right)=\{\begin{array}{c} 0,L_{N}^{\left(i\right)}\left(y_{1}^{N},{\hat{u}}_{1}^{i-1}\right)\geq 0\\ 1,L_{N}^{\left(i\right)}\left(y_{1}^{N},{\hat{u}}_{1}^{i-1}\right)<0 \end{array}$$

where $L_{N}^{\left(i\right)}\left(y_{1}^{N},{\hat{u}}_{1}^{i-1}\right)$ represents the log likelihood ratio of i-th bit, which is

(4) $$L_{N}^{\left(i\right)}\left(y_{1}^{N},{\hat{u}}_{1}^{i-1}\right)=ln\left(\frac{W_{N}^{i}\left(y_{1}^{N},{\hat{u}}_{1}^{i-1}|0\right)}{W_{N}^{i}\left(y_{1}^{N},{\hat{u}}_{1}^{i-1}|1\right)}\right)$$

**Figure 1:**
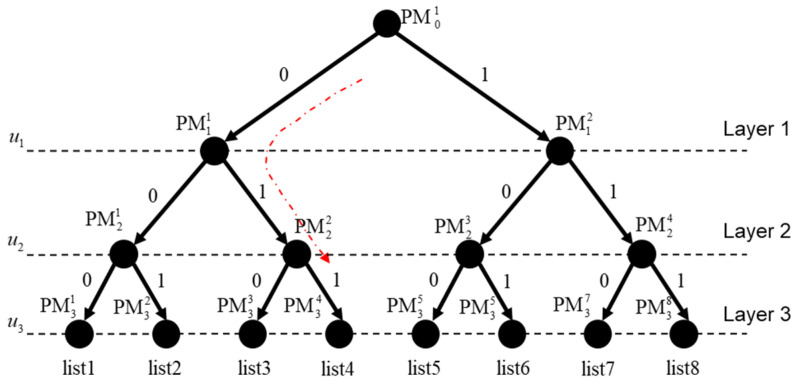
Binary tree of SCL (successive cancellation list) decoding.

It can be seen that the decoding result of the current codeword is related to all previous decoding results in SC decoding, that is, the previous error bit will affect the decoding result of the current bit. In order to solve this problem, the SCL algorithm proposed in [3] expresses all possibilities of SC decoding in the form of a binary tree, as shown in Figure 1. The red dotted line represents a path of list4, and the two branches of each node represent the two possibilities of the node with “1” or “0”. For example, the information bit $u_{1}$ corresponds to the two paths of the first layer in Figure 1. ${\mathrm{PM}}_{1}^{1}$ and ${\mathrm{PM}}_{1}^{2}$ represent the reliability of the two paths, respectively.

## 3. Improved Segmented CRC-Based Adaptive SCL Algorithm

The SCAD-SCL proposed in [21] adds some segmented CRC based on the AD-SCL algorithm, which further reduces the complexity of AD-SCL, but it still has high computation complexity. In our work, we propose an improved algorithm SAD-SCL based on the existing CRC adaptive successive cancellation list algorithm.

The SAD-SCL algorithm introduces segmented CRC for SCAD-SCL and uses a new method to reduce the redundancy calculation of the AD-SCL algorithm. Before each update, the value L is re-decoded, SAD-SCL first uses the existing LLR information to determine the range of the burst error bit, and then only re-decodes the information sequence of this range. Moreover, when SAD-SCL cannot obtain the correct decoding value in the first part of the information sequence, it uses SC decoding to complete the decoding in the second part of the information sequence, and the decoding performance of SAD-SCL is almost the same as that of second part of the AD-SCL algorithm. This paper introduces two improvements to SAD-SCL in Section 3.1 and Section 3.2. The specific decoding process for SAD-SCL is given in Section 3.2.

### 3.1. Adaptive SCL Decoding Based on Burst Error Bits

**Figure 2:**
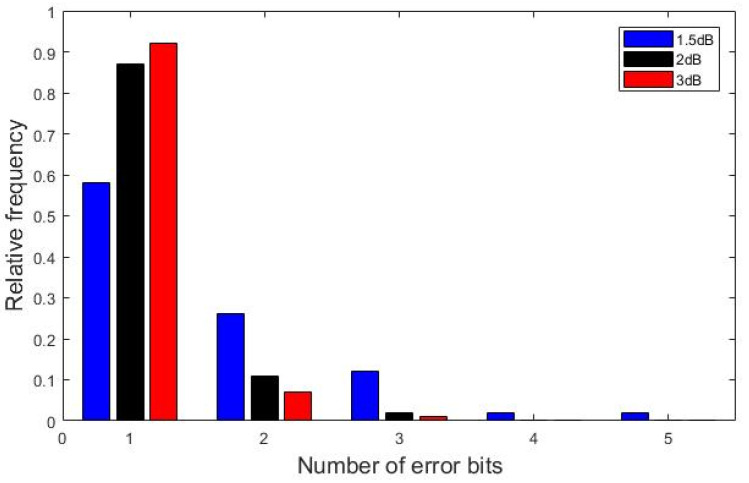
The number of error bits for different SNR at *N* = 1024, *R* = 0.5.

The error bits of the SC decoding algorithm can be divided into two categories. One is burst error caused by channel interference, and the other is error propagation caused by burst error bits. In [22], an SC decoding algorithm which eliminates inter-symbol interference is designed to count the number of burst error bits generated by channel interference. When code length is N=1024 and code rate is R=0.5, Figure 2 illustrates the number of error bits counted for 500,000 data frames for different signal to noise ratio (SNR) by using the experimental method designed in [22,23].

**Figure 3:**
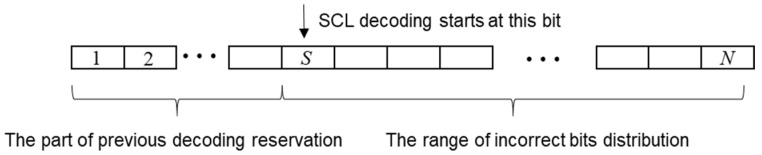
SCL decoding structure diagram starting from S.

It can be seen that the burst error bits of the SC decoding are mostly within three bits, and the probability of only one burst error bit is the largest. Therefore, if the decoding result does not pass the CRC, there is a great possibility caused by a burst error bit of information. We can select an appropriate information bit $i_{s}$, which is located in front of all the error bits. When re-decoding, the decoded bits before the S-th bit are reserved, and the information bits after the S-th bit are decoded by using the increasing L decoding algorithm. A schematic diagram of the process is shown in Figure 3.

In the decoding process, the larger the absolute value of LLR of an information bit is, the more reliable the decision value of the information bit is. Let R be the set of values LLR of the decoded information bits and frozen bits in SCL, and I is the set of indexes in *R* in the original information sequence. Based on this feature, we can find a suitable value by following these steps:

Step 1: When the decoding of the segmented CRC fails, we find the m bits with smaller absolute LLR values in set R and record the indexes as a set $I_{e}$.

Step 2: Let S be the smallest index in set $I_{e}$.

In this paper, the above method is used to dynamically find a position S in the information sequence, so that all possible burst error bits are located at the right of S (assuming the decoding order is from left to right), and the SCL decoding is restarted from this bit. It ensures that SCL contains all burst error bits. Compared with the SCAD-SCL and AD-SCL algorithms, the SAD-SCL makes full use of previous decoding results and digs out the available information, which reduces redundant calculation. From the above analysis, the larger the value m is, the higher the probability of burst error bits contained in the set $I_{e}$ will be, but the position of S will be closer to the starting bit of the decoding sequence, which means that this SCL decoding will calculate more nodes. In addition, if the value m is too small, the burst error bit may be missed in the set $I_{e}$, which will result in loss of decoding performance. The value m will be analyzed in detail in Section 4.

### 3.2. Improved Algorithm Based on Segmented Adaptive SCL

Since SC decoding has error propagation characteristics, the number of decoded error bits will be very large when errors occur. SCL also shares a similar phenomenon. In [24], there are two types of errors in SCL, which can be divided into disappearing errors and selection errors. Disappearing errors are when the correct paths are not included in the L paths of SCL. Selection errors are when error paths are selected from the L paths which include correct paths, because the value PM of correct paths is not enough to be selected. When the decoding has disappearing errors, the correct paths will never be selected after the decoding of this bit.

In the SCAD-SCL algorithm, if the first part using SCL decoding with the parameter $L=L_{\max}$ fail to pass CRC, it means that the correct decoding result is not included in the L paths in the first part. The decoding error at this time is a disappearing error. The decision of SCAD-SCL is to take the optimal path as an output, and then the algorithm begins the next part of adaptive SCL decoding. However, the decoding of the latter part is based on the previous part, no matter how large L is, SCL cannot decode the correct codes of the latter part. In addition, due to the failure of the CRC, the adaptive SCL decoding of this segment will increase from $L_{\min}$ to $L_{\max}$, which greatly increases delay and complexity. Thus, the decoding of the latter part under this situation is completely unnecessary.

The SAD-SCL algorithm proposed in this paper makes full use of the above analysis and improves the existing SCAD-SCL algorithm in two aspects.

Improvement 1: The SAD-SCL algorithm uses the method described in 3.1 to find the value S before each update value L is re-decoded and takes *S* as the starting bit for decoding.

Improvement 2: When the SAD-SCL algorithm cannot get the correct decoding result of the first part, the SAD-SCL algorithm directly uses SC decoding to complete the decoding of the second part.

**Figure 4:**
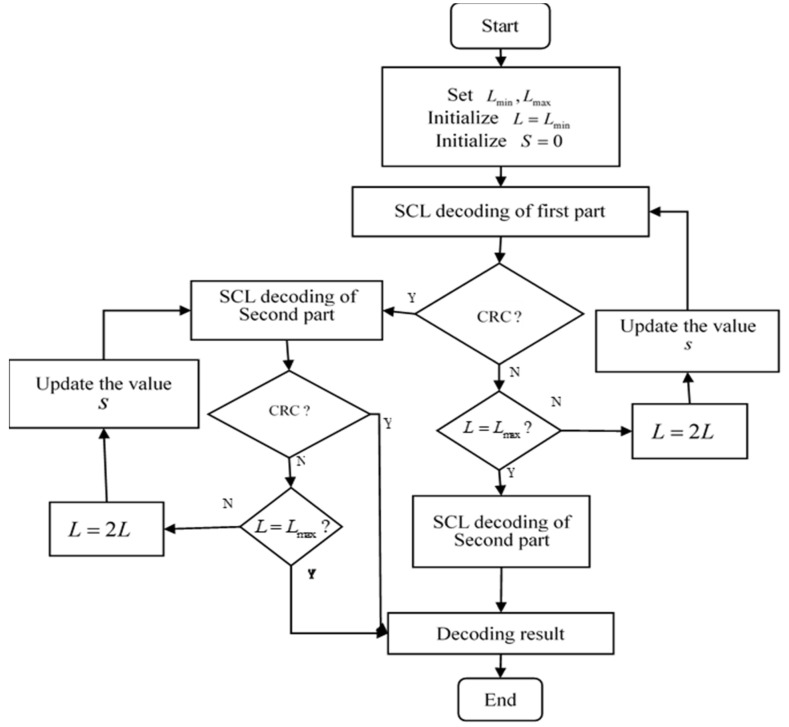
SAD-SCL decoding process.

The flow chart of the SAD-SCL decoding algorithm is shown in Figure 4. Let $\hat{u}$ denote the decoding output value of SAD-SCL. The decoding algorithm can be divided into the following decoding steps:

Step 1: L is set as $L_{\min}=1$, then ${\hat{u}}_{1}^{1}$ and $R_{1}^{1}$ are obtained after the SCL decoding starts in the first part, where ${\hat{u}}_{1}^{1}$ represents the SCL decoding result of the first part, and $R_{1}^{1}$ is the value LLR corresponding to ${\hat{u}}_{1}^{1}$.

Step 2: If the CRC of ${\hat{u}}_{1}^{1}$ fails, then the decoding process of the first part begins to re-decode by the improved adaptive SCL algorithm. The decoder makes a comparison between L and $L_{\max}$. If L is less than $L_{\max}$, then sets L=2L, and calculates S according to $R_{1}^{1}$, where S indicates the starting bit of re-decoding. Then ${\hat{u}}_{1}^{2}$ and $R_{1}^{2}$ are obtained by re-decoding, if the CRC of ${\hat{u}}_{1}^{2}$ fails, continue to increase *L* and update the value *S*, then ${\hat{u}}_{1}^{3}$ and $R_{1}^{3}$ are obtained by re-decoding and so on, the algorithm goes to step 3 until the CRC of ${\hat{u}}_{1}^{t_{1}}$ passes($0<t_{1}\leq log_{2}L_{max}$,where $t_{1}$ denotes the number of re-decoding of the first part). In addition, when $L=L_{\max}$, if the decoding result still fails to pass CRC, the first part of the codes is judged to include error bits, and the decoder goes directly to step 4.

Step 3: SAD-SCL begins the decoding of the second part, the decoding result and its corresponding LLR values of second part are ${\hat{u}}_{2}^{t_{2}}$ and $R_{2}^{t_{2}}$ ($0<t_{2}\leq log_{2}L_{max}$, where $t_{2}$ denotes the number of re-decoding of the second part), respectively. The decoding process of the second part is the same as that of the improved adaptive SCL in step 2. However, in the decoding of the second part, when $L=L_{\max}$, the decoder directly outputs the final decoding result $\hat{u}=\left\{{\hat{u}}_{1}^{t_{1}},{\hat{u}}_{1}^{t_{2}}\right\}$ if the decoding result of ${\hat{u}}_{2}^{t_{2}}$ does not pass CRC.

Step 4: Since there are some error bits in this decoding result, the SC algorithm is directly used for the decoding of the second part. Assume the result of the decoding is ${\hat{u}}_{2}$, then the final output decoding result is $\hat{u}=\left\{{\hat{u}}_{1}^{t_{1}},{\hat{u}}_{2}\right\}$.

**Figure 5:**
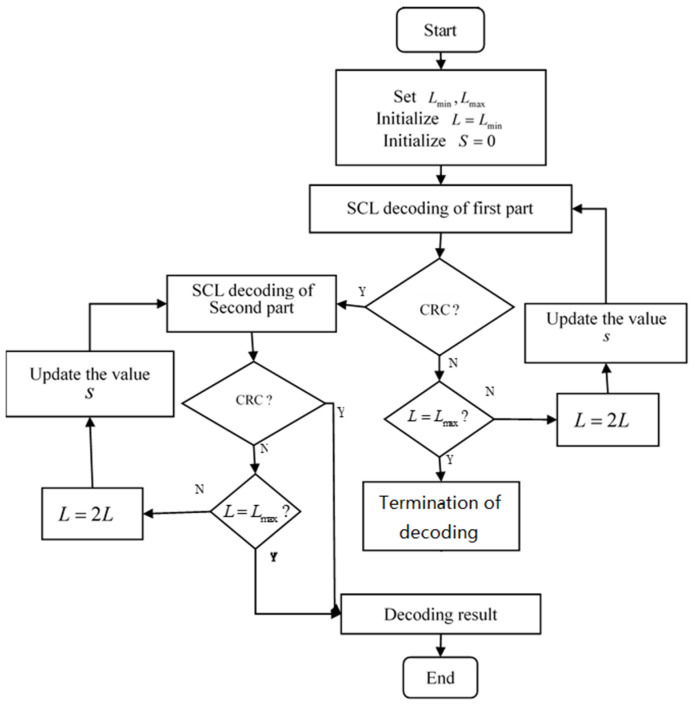
Modified SAD-SCL decoding process.

Considering the FER performance of the algorithm, the proposed algorithm can be further improved. If the first part of decoding fails to pass CRC with $L=L_{max}$, then the whole codeword will be in error. When the decoding is terminated, the number of error frames can also be counted. Therefore, the termination of the second part of decoding will not lose FER performance. So, the second part of decoding is unnecessary in this situation. This decoding can be terminated to avoid wasting resources. This can reduce the complexity of decoding without loss of performance. The modified algorithm decoding process is shown in Figure 5.

## 4. Experiment and Analysis

An adaptive segmented SCL decoding algorithm uses S as the starting bit of re-decoding in this experiment. In order to measure the computation complexity of the decoding algorithm, the average number of calculations of PM value proposed in [21] is used. The segmented method is to divide the bits of information into two parts and decode them respectively, which is same as that in the SCAD-SCL algorithm. When the code length and code rate are 1024 and 0.5, respectively, the number of information bits are 512, the last 8-bit information of each segmentation are CRC bits. After adding the frozen bits, there are 748 bits at the first part of the information sequence and just 276 bits at the second part of the information sequence. There are 492 frozen bits at the first part while there are only 20 frozen bits at the second part. Because the second part of information sequence is shorter and contains more intensive information bits, the computational complexity reduction caused by adding S is not enough to compensate for its performance loss. Therefore, this experiment only adds S at the first 748 bits of information sequence.

In order to observe the change of computational complexity under different values of m more intuitively, the average number of calculations of PM values are counted when the decoding starts at S-th bit. The experimental parameters are shown in Table 1.

**Figure 6:**
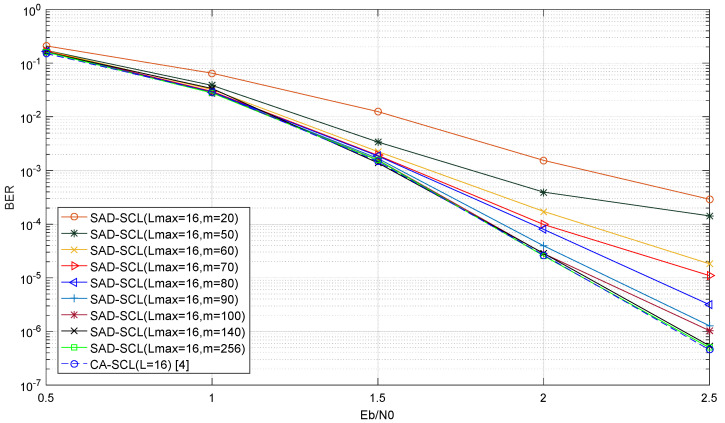
BER Performance of SCL at L=16 under different values of m.

**Figure 7:**
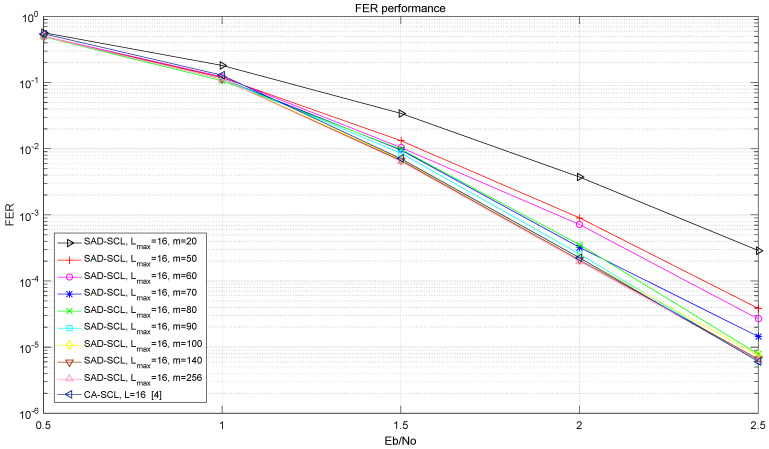
FER Performance of SCL at L=16 under different values of m.

Figure 6 shows the bit error rate (BER) performance curves of SAD-SCL ($L_{max}$) and CA-SCL (L=16) with a code length of 1024 and a code rate of 0.5 under AWGN channel and BPSK modulation. Figure 7 shows the frame error rate (FER) performance curves of SAD-SCL ($L_{max}$) and CA-SCL (L=16) under the same condition as Figure 6. As can be seen from Figure 6 and Figure 7, with the increase of m, the performance of adaptive algorithm designed by this experiment improves continuously. The performance of this algorithm is almost the same with that of SCL when m=140, which illustrates that $R_{e}$ contains almost all burst error bits. Table 2 shows the average number of calculations of PM value when m takes different values. It can be seen in Table 2 that the smaller the value of m, the smaller the average number of calculations of PM value. But the performance decreases as the value of m reduces, which indicates that a smaller value of m will make $R_{e}$ more likely to miss burst error bits. Taking performance and computational complexity into account, m=140 is used as the parameter of SAD-SCL decoding algorithm.

**Figure 8:**
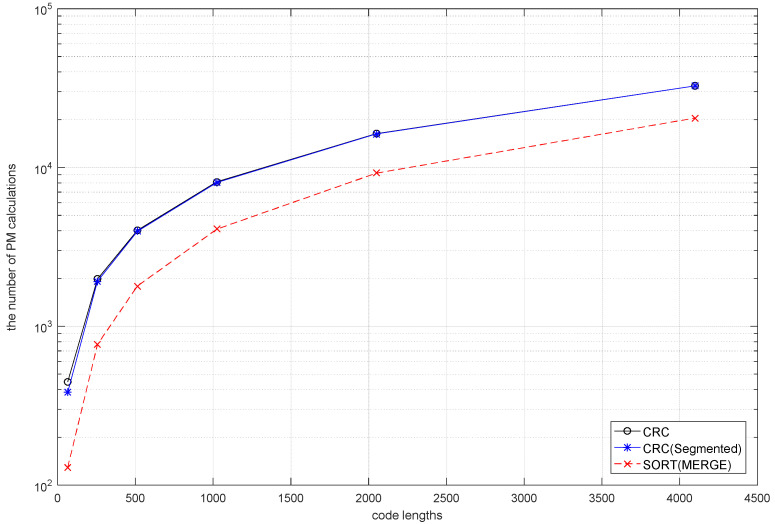
The number of PM calculations for CRC, two-segmentation CRC and merge sort of N/2-bits under different code lengths.

When the code length, the number of information bits and the length of CRC are N, K, M, respectively, the AD-SCL algorithm needs to consume additional (N−M)×M times of addition calculation for CRC every decoding, while the SCAD-SCL algorithm and the SAD-SCL algorithm proposed in this paper need additional (N/2−M)×M×2 times of addition for CRC each time. In addition, the SAD-SCL algorithm proposed in this paper needs a merge sorting of N/2-bits when choosing the starting point S of re-decoding. The merge sorting requires at most $\left(K/2\right)\log_{2}\left(K/2\right)-K+1$ additional calculations. Figure 8 shows the number of addition calculation times of CRC, two-segmentation CRC and merge sorting algorithms when the code rate is 0.5 and the code lengths are 64, 512, 1024, 2048, 4096.

It can be observed that the computation complexity of CRC and two-segmentation CRC is almost the same whatever the code length is, and the difference between them can be ignored. Compared with CRC and two-segmentation CRC, there exists an extra N/2 merge sort in SAD-SCL.

In [12], the calculation of CRC is almost negligible compared with the average number of calculations of value PM (LLR value, etc.) each time in SCL. Therefore, the computation resource consumed by merge sort can be ignored in SCL. Compared with the SCAD-SCL and AD-SCL algorithms, SAD-SCL makes full use of previous decoding results and digs out the available information, which reduces redundant calculations.

**Figure 9:**
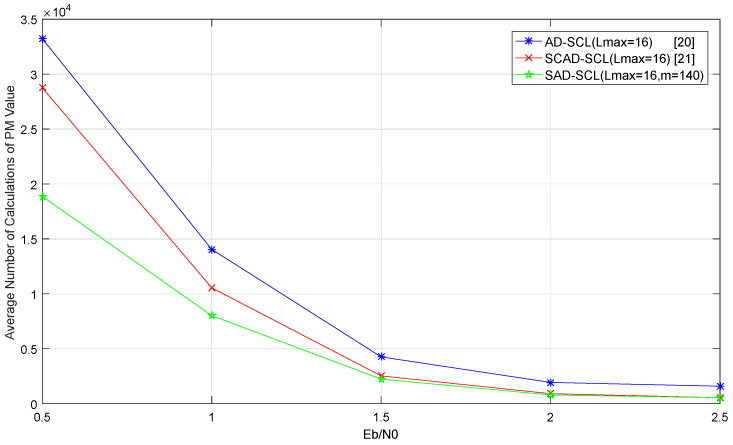
The average number of calculations of value PM for AD-SCL, SAD-SCL, SCAD-SCL with $L_{max}=16$.

When the code length is 1024 and the code rate is 0.5 respectively, the average number of calculations of value PM for AD-SCL, SCAD-SCL, SAD-SCL with $L_{max}$ = 16 is shown in Figure 9. It is observed that the complexity of the three adaptive algorithms declines with the increase of SNR.

The reason is that there are more error frames caused by inter-symbol error propagation in low SNR and the adaptive algorithm needs a large value of L to complete the decoding. With the increase of SNR, the frame number of inter-symbol error propagation decreases gradually, and less values of *L* are required for the adaptive algorithm, so the complexity in high SNR is lower than that in low SNR. In addition, the computation complexity of the SAD-SCL algorithm proposed in this paper is lower than AD-SCL and SCAD-SCL. But with the increase of SNR, the adaptive algorithm tends to complete decoding once. At this time, the complexity of three kinds of algorithms is almost the same. In conclusion, the gap of complexity of three kinds of algorithms will narrow with the increase of SNR.

**Figure 10:**
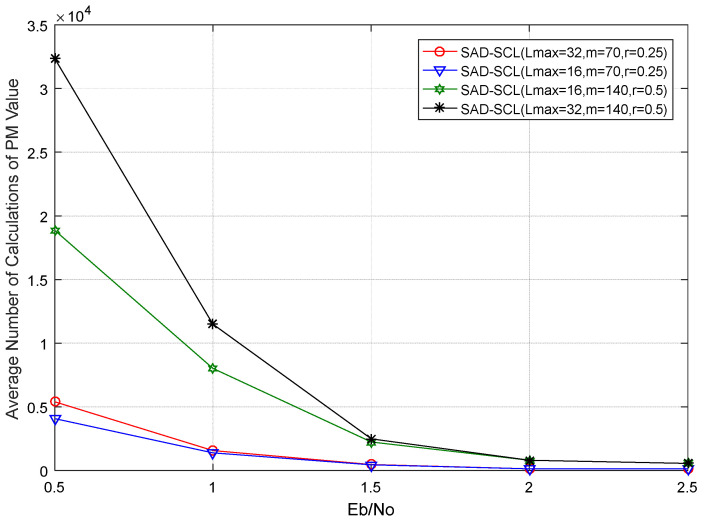
Average PM update times of SAD-SCL algorithm under different $L_{max}$ and *m*.

Figure 10 shows the comparison curves of the average number of calculations of PM value for SAD-SCL algorithm under different code rates and $L_{max}$. It can be seen that the smaller the code rate and L are, the less the average PM update times of SAD-SCL are.

**Figure 11:**
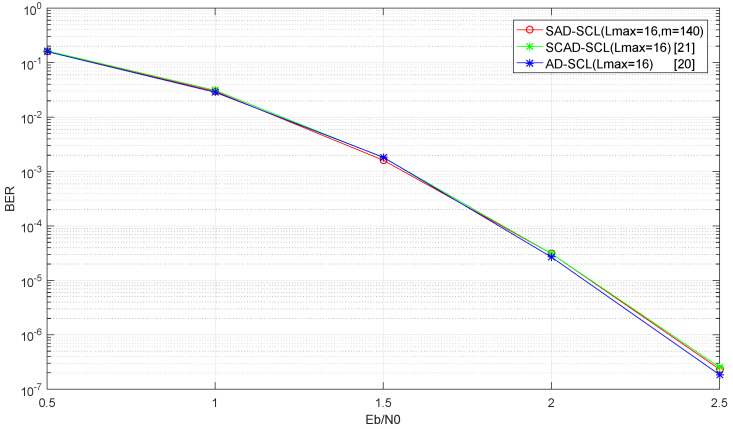
Performance curve of BER about SAD-SCL, SCAD-SCL, CA-SCL with $L_{max}=16$.

**Figure 12:**
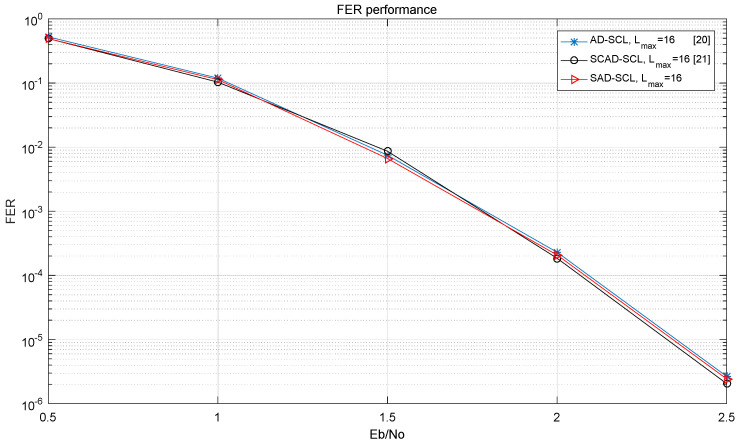
Performance curve of FER for SAD-SCL, SCAD-SCL, CA-SCL with $L_{max}=16$.

Figure 11 and Figure 12 show the BER and FER performance curves of the SAD-SCL (*m* = 140), SCAD-SCL, AD-SCL algorithms when $L_{max}$ = 16 under AWGN channel and BPSK modulation. It is observed that there is little performance loss from the SAD-SCL algorithm compared to the other two adaptive algorithms.

## 5. Conclusion

In order to overcome the shortcomings of the existing adaptive SCL algorithm, this paper proposes an improved SAD-SCL algorithm, which is from the idea of segmented CRC of SCAD-SCL. The algorithm uses a new method to reduce the complexity of the AD-SCL algorithm. Before the re-decoding of updating value L each time, SAD-SCL first uses the existing LLR information to detect the range of burst error bits, and then decode the information sequence of this range again. Moreover, when SAD-SCL cannot get the correct decoding value in the first part of the information sequence, it uses SC decoding with less computation to complete the decoding in the second part of the information sequence. Its decoding performance is almost the same as that in the second part of the information sequence using the AD-SCL algorithm. The simulation results show that the SAD-SCL algorithm has lower computational complexity than AD-SCL and SCAD-SCL without loss of performance.

## Figures and Tables

**Table 1:** Experiment parameter.

Category	Parameter
Encoding	CRC and polar code
Channel type	AWGN
Code length	1024
L (the size of list)	$L_{min}$, $L_{max}$
Code rate	0.5
modulation method	BPSK
Decoding	Adaptive segmented Decoding Algorithm for AD-SCL with S as the starting bit of re-decoding
m (the number of LLR)	20, 50, 70, 80, 90, 100, 140, 256

**Table 2:** Calculation times of average PM under different values of m.

m	Eb/N0=0.5	Eb/N0=1	Eb/N0=1.5	Eb/N0=2	Eb/N0=2.5
	PM	PM	PM	PM	PM
50	16402	6548	1436	218	32
80	16692	6199	1486	218	25
100	16962	6352	1497	220	26
140	17277	7132	1580	228	28
256	19076	7983	1724	261	32
